# Supplementary material for: Effectiveness of routine tuberculosis education in a high-burden setting: A prospective observational cohort study
Source: PLoS One. 2026 Mar 18;21(3):e0344250. doi: 10.1371/journal.pone.0344250 (PMC12998860; doi:10.1371/journal.pone.0344250)
Supplement: S1 Appendix — (PDF) [file pone.0344250.s001.pdf]

S1 Appendix. Participant Questionnaire

<u>Respondent Information</u>

1. Treatment initiation date
2. NTLP Register Number
3. Sex
4. Date of birth
5. What is the highest level of education you have completed?
6. What is your current occupation?
7. Referred to the TB unit from…
8. Did you receive TEC before coming to the TB unit for this TB episode?
9. What is your HIV status?
10. Have you ever had TB before?
11. Has someone in your household or anyone close to you had TB?
12. Have you ever received any information about TB?
    a. If yes, where have you received information about TB?
        i. Religious leaders (Yes/No)
        ii. Health workers (Yes/No)
        iii. Family and friends (Yes/No)
        iv. Teachers (Yes/No)
        v. Brochures, posters or other printed materials (Yes/No)
        vi. Media (Yes/No)

<u>TB Knowledge: Closed-Ended Questions</u> *<u>(Construct in parentheses)</u>*

1. TB attacks the lungs. *(TB Basics)*
2. TB can attack other parts of the body outside of the lungs. *(TB Basics)*
3. Everyone who is exposed to TB germs becomes ill. *(TB Basics)*
4. Answer "yes" if the condition is a warning sign of TB and answer "no" if it is not a warning sign of TB. *(TB Symptoms)*
    a. A cough that does not go away for two weeks
    b. Loss of weight
    c. A cough that goes away after a few days
    d. General weakness
    e. Vomiting
5. Answer "yes" if the statement is a way that TB can be spread and answer "no" if TB cannot be spread that way. *(TB Transmission)*
    a. Through drinking water
    b. Through the air
    c. Through food
6. Unprotected sex can spread TB. *(HIV-TB Relationship)*
7. If you stop treatment before the full course of therapy, your TB becomes harder to cure. *(TB Treatment Principles)*

TB Knowledge: Open-Ended Questions + Decision Logic (*Content Domain in parentheses)*

1. How often can TB be cured if treatment is started in time? *(TB Treatment Principles)*
    a. Correct (always, almost always, "I am pretty sure TB can be cured if you complete treatment")
    b. Incorrect (never, sometimes, doesn't know)
2. How are TB germs released? *(TB Transmission)*
    a. Correct (when a person with TB coughs, sneezes, spits)
    b. Incorrect (when a person with TB vomits, touches you; if you share a cup or food with someone who has TB; doesn't know)
3. If you breathe in TB germs, where do they settle and grow? *(TB Transmission)*
    a. Correct (lungs)
    b. Incorrect (anywhere else, doesn't know)
4. Can you have HIV only (without TB)? *(HIV-TB Relationship)*
    a. Correct (yes)
    b. Incorrect (no, doesn't know)
5. Can you have TB only (without HIV)? *(HIV-TB Relationship)*
    a. Correct (yes)
    b. Incorrect (no, doesn't know)
6. How can you stop the spread of TB? *(TB Prevention)*
    a. Correct (cover mouth with a handkerchief; cover mouth when coughing; avoid others for the first two weeks of treatment; don't go out in public for the first two weeks of treatment; don't spit on the ground in public)
    b. Incorrect (tell everyone that I have TB; interviewer uses judgement; doesn't know)
7. If you have TB, how long do you take the medication? *(TB Treatment Regimen)*
    a. Correct (until you are discharged, 6-8 months; when they test me again and there is no more TB and the nurse tells me I can finish treatment)
    b. Incorrect (any other answer, doesn't know)
8. When can you stop taking the TB medication? *(TB Treatment Principles)*
    a. Correct (when you finish the full course of therapy, when you are discharged, after 6-8 months)
    b. Incorrect (when the cough stops, when you feel better, doesn't know
9. If you stop taking the TB medication before the treatment period is finished, what might happen? *(TB Treatment Principles)*
    a. Correct (the germs won't be fully killed and another TB episode can occur; the germs can develop resistance; you can die; you can end up taking the medication for a longer period of time)
    b. Incorrect (nothing, doesn't know)
10. Name two potential side effects of TB treatment. *(TB Treatment Regimen)*

a. Correct (skin rash, nausea, joint pain, color of urine changes, etc.—interviewer uses judgment)
b. Incorrect (interviewer uses judgment; can only name one; doesn't know)

11. What should you do if your TB medication gives you nausea? *(TB Treatment Regimen)*
    a. Correct (endure for a few weeks, try taking medication with food or other self-medication such as lemon)
    b. Incorrect (stop taking medication, come to the clinic, doesn't know)
12. What should you do if your TB medication gives you joint pain? *(TB Treatment Regimen)*
    a. Correct (take pain killers, take pyridoxine, move around and do some light exercises, reduce on doing heavy work)
    b. Incorrect (stop taking medication, come to the clinic, doesn't know)
13. What should you do if your TB medication gives you yellow or red eyes, too much vomiting, intense body rash, or issues with sight? *(TB Treatment Regimen)*
    a. Correct (talk to a health worker, come to the clinic)
    b. Incorrect (stop taking medication, sleep it off, nothing, doesn't know)
14. When should you come to the clinic for your next appointment? *(TB Treatment Follow-up)*
    a. Correct (be able to say the date or the amount of time until the next appointment)
    b. Incorrect (when the health worker tells me to come back; can't tell the date or the amount of time until the next appointment; doesn't know)
15. What do your TB medications look like? *(TB Treatment Regimen)*
    a. Correct (adequately explains what pills look like in color and shape)
    b. Incorrect (incorrectly explains what the pills look like; doesn't know)
16. When do you take your TB medications? *(TB Treatment Principles)*
    a. Correct (in the morning when you first wake up)
    b. Incorrect (any other time; multiple times throughout the day; doesn't know)
17. After taking the medication, how long does it usually take to start feeling better? *(TB Treatment Follow-up)*
    a. Correct (any amount of time between 2 weeks and 1 month)
    b. Incorrect (any time outside of 2 weeks and 1 month; doesn't know)

<u>Self-Reported Nonadherence</u>

1. During the last week, how many days were you late or missed taking your TB medication?
2. Below are some reasons why people have difficulty taking their drugs. Answer "yes" or "no" to indicate whether or not each of the following reasons describes why you may have had difficulty taking your drugs in the last 7 days.
    a. I feel worse when I take the pills
    b. There are too many pills to take

c. I forget to take the pills
d. I ran out of pills
e. I don’t think I need the pills
f. I was away from home
g. I did not want others to notice
h. I am too busy
i. I had problems taking pills at specified times
j. I was confused or uncertain about how to take the pills
