# Supplementary material for: Effectiveness of routine tuberculosis education in a high-burden setting: A prospective observational cohort study
Source: PLoS One. 2026 Mar 18;21(3):e0344250. doi: 10.1371/journal.pone.0344250 (PMC12998860; doi:10.1371/journal.pone.0344250)
Supplement: S1 File — (PDF) [file pone.0344250.s002.pdf]

TB and HIV Health Education Flipchart

UGANDANS AND AMERICANS IN PARTNERSHIP TO FIGHT HIV/AIDS

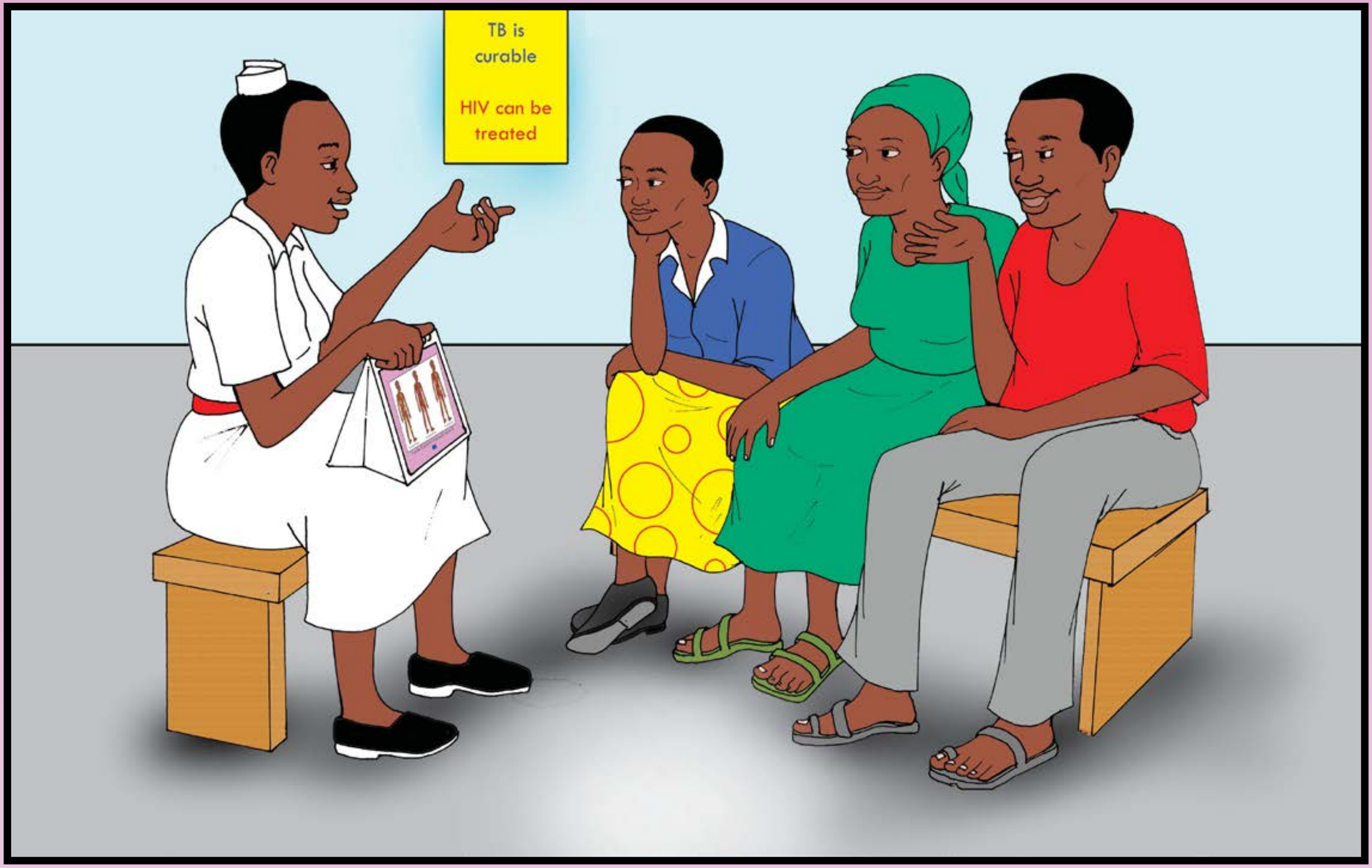

THE REPUBLIC OF UGANDA
MINISTRY OF HEALTH

TB and HIV Counseling Flipchart

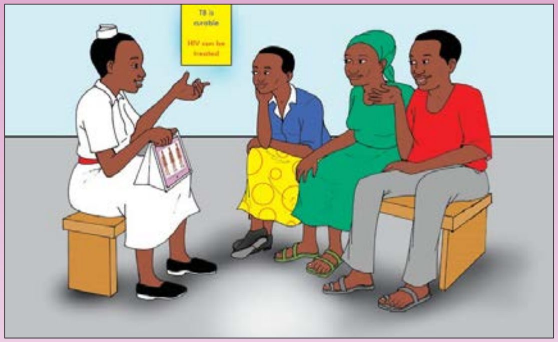

TB and HIV Counseling Flipchart

This flipchart is meant to be used by health workers. It is designed for counseling patients who have tuberculosis (TB) or those who have HIV.

Charts included:

1. *What is TB?*
2. *What are the warning signs of TB?*
3. *How is TB spread?*
4. *TB and HIV*
5. *TB, HIV and your immune system*
6. *Where do you go for TB and HIV testing?*
7. *How do you test for TB?*
8. *Complete your TB treatment*
9. *What are the side effects of treatment?*
10. *How do you prevent the spread of TB?*
11. *Continue with ARVs after TB treatment*

The flipchart has three main objectives:

❶ to provide information about TB and HIV

❷ to encourage people living with HIV to test for TB and, if necessary, and get treatment

❸ to encourage people living with TB to test for HIV and, if necessary, and get treatment

This flipchart is useful in one-on-one sessions with patients as well as small group discussions.

How to use the flipchart:

- Welcome your patient and sit face to face.
- While you use the side with text, make sure the patient has a good view of the pictures.
- Point to the picture while you speak.
- Speak clearly and use language that the patient can understand.
- Avoid reading the text.
- Try to involve the patient by asking questions. Review questions are included at the end of each page to assist you.

What is TB?

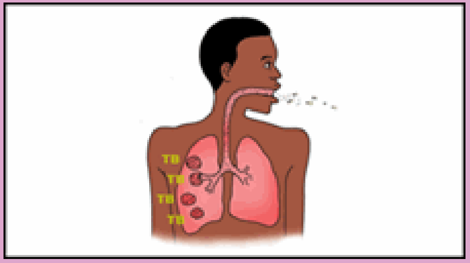

What are the warning signs of TB?

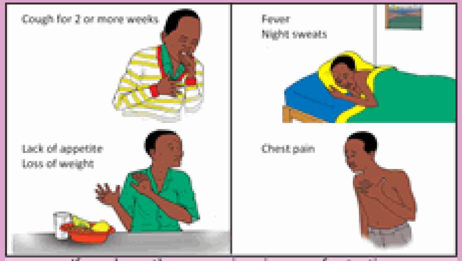

If you have these warning signs, go for testing

How is TB Spread?

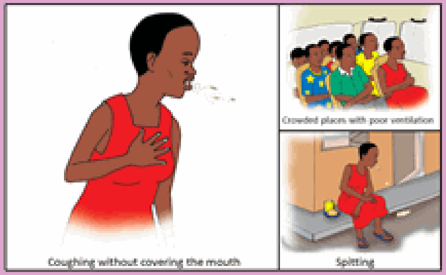

TB and HIV

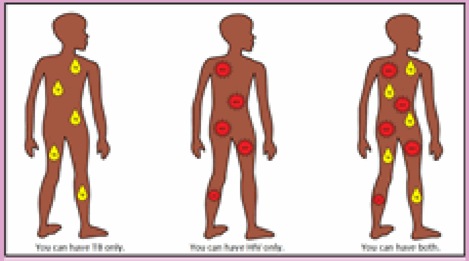

If you have TB, test for HIV. If you have HIV, test for TB.

TB, HIV and your immune system

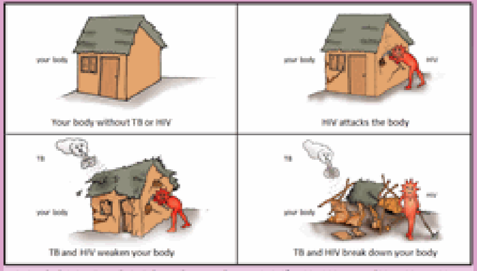

Don't let TB and HIV break you down. Go for testing and treatment.

Where do you go for TB and HIV testing?

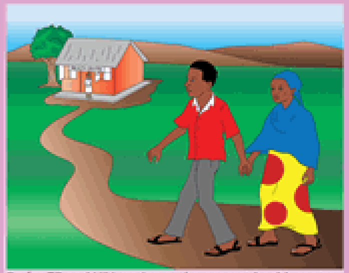

Go for TB and HIV testing at the nearest health centre.

How do you test for TB and HIV?

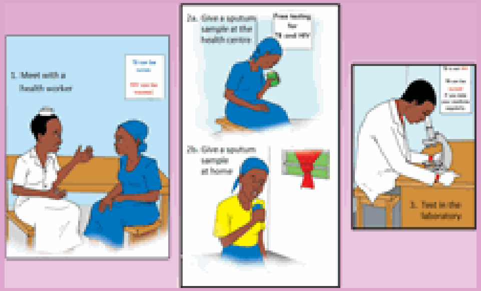

Complete your TB treatment

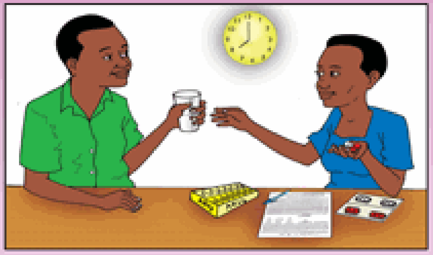

Take all your TB and HIV drugs on time each day.

What are the side effects of treatment?

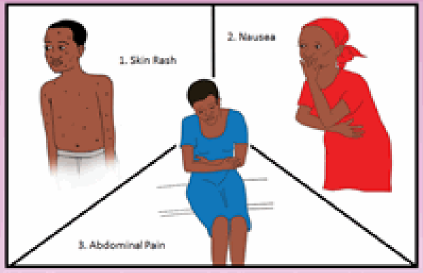

Side effects can be managed. Tell your doctor right away.

How do you prevent the spread of TB?

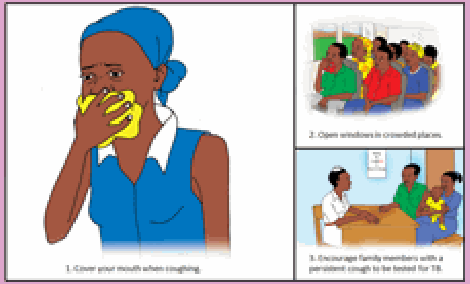

Continue with ARVs after TB is cured

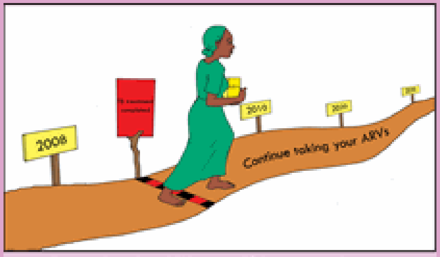

Remember that ARVs are a lifelong commitment.

What is TB?

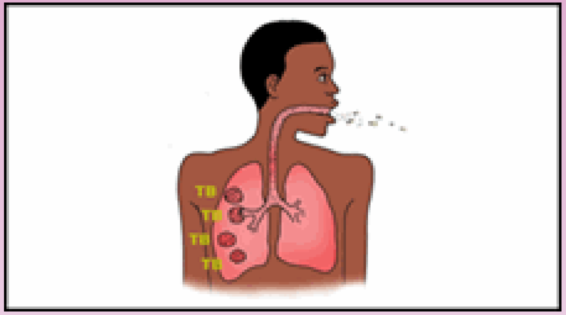

What is TB?

OBJECTIVE:

To describe the TB disease

Notes to the health worker

- *Encourage the patient to share their personal TB knowledge and experiences with you.*
- *Confirm the correct information they share with you. Identify and correct any wrong information.*

TB is a disease caused by a germ that attacks the lungs. It can also attack other parts of your body such as your bones, joints and intestines.

TB is spread from one person to another through the air.

You can have TB and have no symptoms for some time. This is “silent” TB. When TB becomes “active” you will have a cough that lasts for more than 2 weeks or symptoms in other parts of your body.

TB can be cured if it is treated properly. If TB is not treated properly, it can lead to death. TB drugs are free in all health facilities in Uganda.

Review Question:

What do you know about TB?

What is TB?

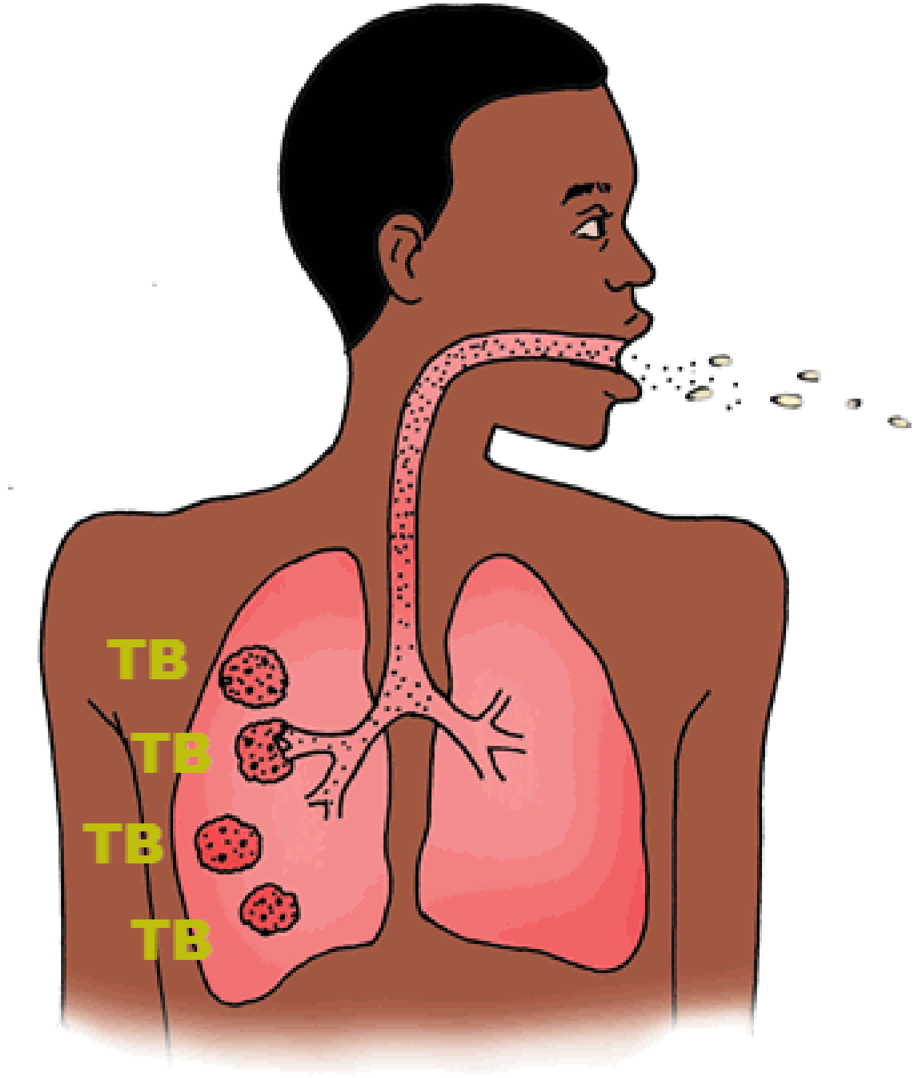

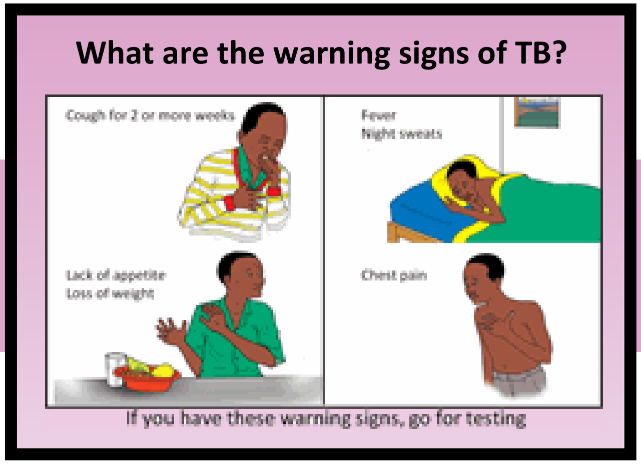

Notes to the health worker

- *Help the patients identify if they or their family members have had any warning signs of TB.*

What are the warning signs of TB?

OBJECTIVE:

To provide information about the warning signs of TB

People with TB demonstrate certain warning signs. They may include one or several of the following:

- A cough that does not go away for 2 weeks or more
- Fever
- Night sweats
- Lack of appetite
- Loss of weight
- Chest pain
- Difficulty breathing
- General weakness
- Coughing up sputum that is stained with blood

It is important for people who have coughed for 2 or more weeks to be tested for TB at a health facility.

Since TB spreads through the air, people with warning signs should go for testing to prevent spreading TB to others.

Review Question:

Can you describe some warning signs of TB?

What are the warning signs of TB?

Cough for 2 or more weeks

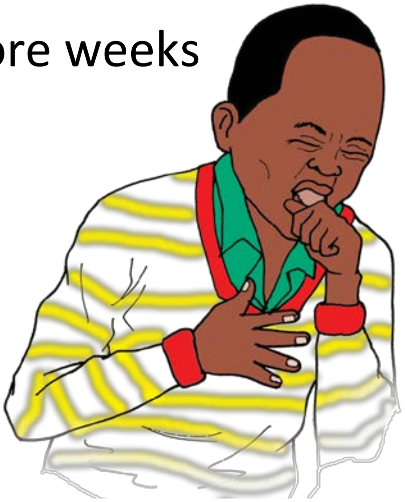

Fever
Night sweats

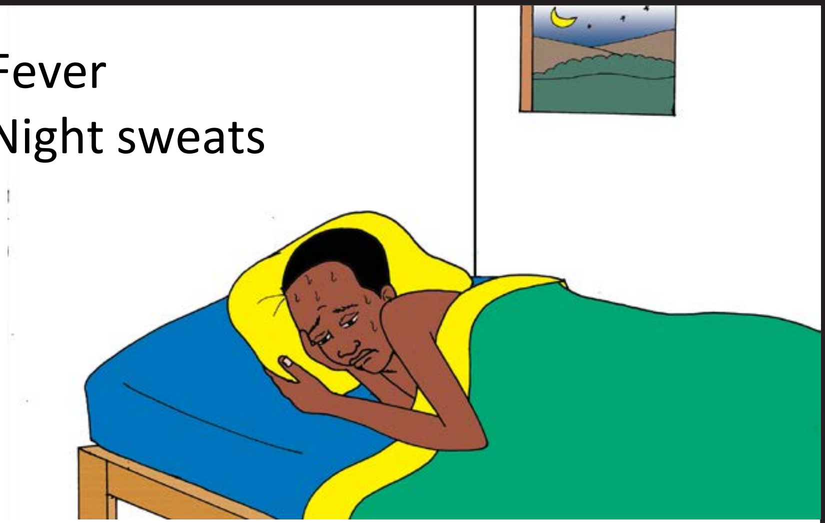

Lack of appetite
Loss of weight

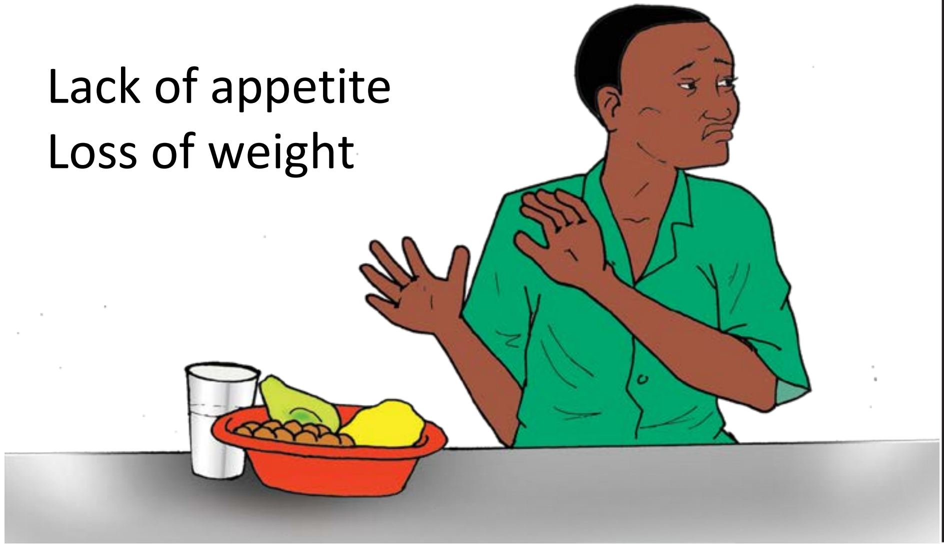

Chest pain

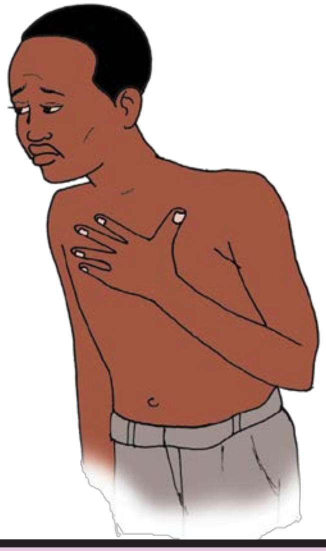

If you have these warning signs, go for testing

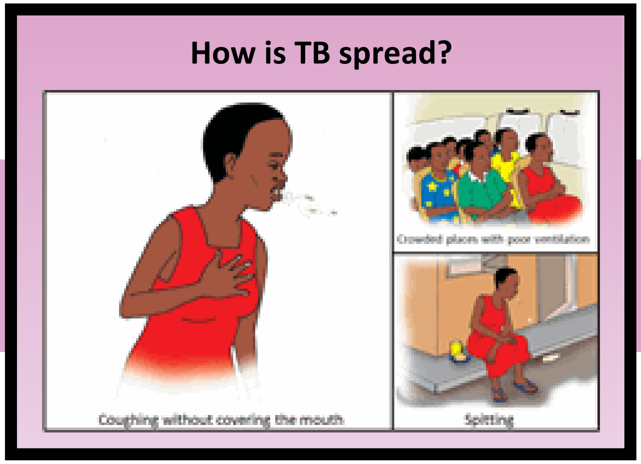

Notes to the health worker

Remember: HIV is spread in three main ways:

1. *By having unprotected sex with a person who has HIV*
2. *By sharing needles or other sharp instruments with a person who has HIV*
3. *From a mother who has HIV to her child during pregnancy, childbirth or through breastfeeding*

How is TB spread?

OBJECTIVE:

To help the patient understand how TB is spread

TB is spread through the air from one person to another.

When a person with TB coughs or sneezes, the TB germs are released. If you breathe in those germs, they can settle in your lungs and begin to grow. From there, they can move through your blood to other parts of your body, such as the kidneys, spine or brain.

How is TB spread? The picture illustrates the following:

1. Coughing or sneezing without covering the mouth
2. Crowded places with poor ventilation
3. Spitting

Other behaviors that spread TB include:

- Kissing a person who has TB

People with TB of the lungs are likely to spread it to those they spend a lot of time with, like family members, friends, classmates, and co-workers.

Remember: TB is not HIV. The way that TB is spread is very different from the way HIV is spread.

Review Question:

Describe how TB is spread. How is HIV spread?

How is TB Spread?

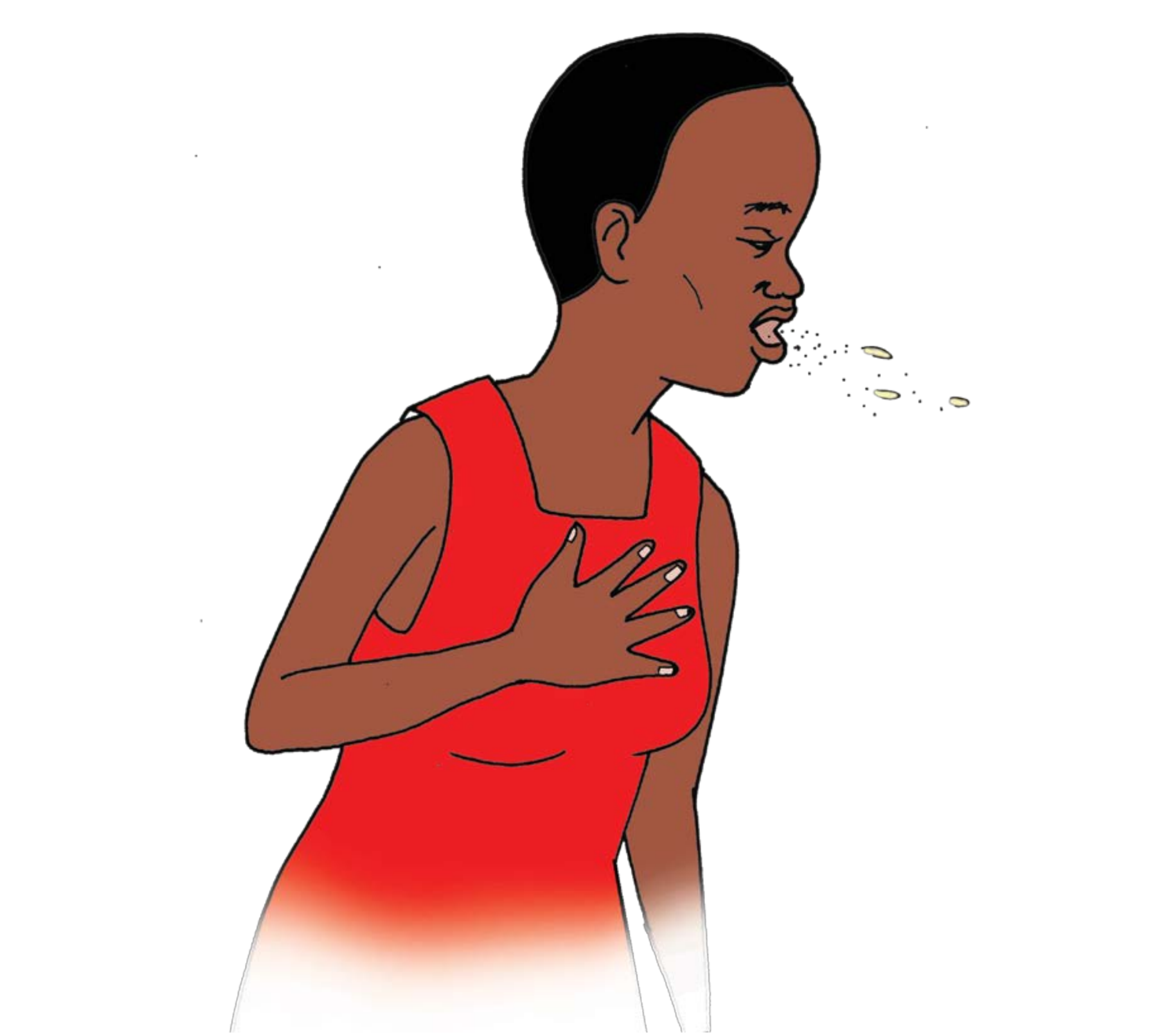

Coughing without covering the mouth

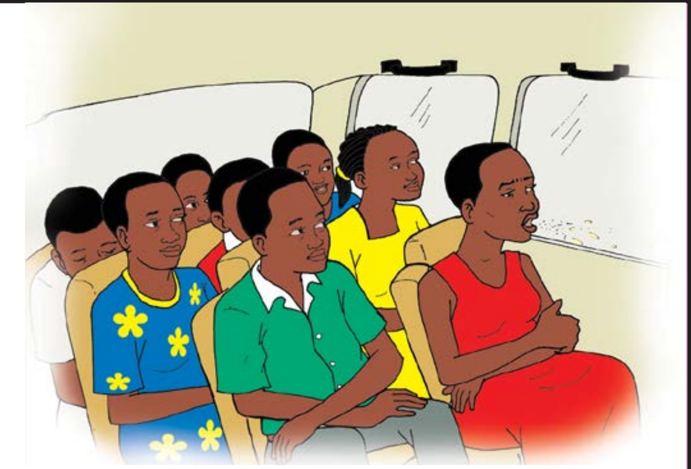

Crowded places with poor ventilation

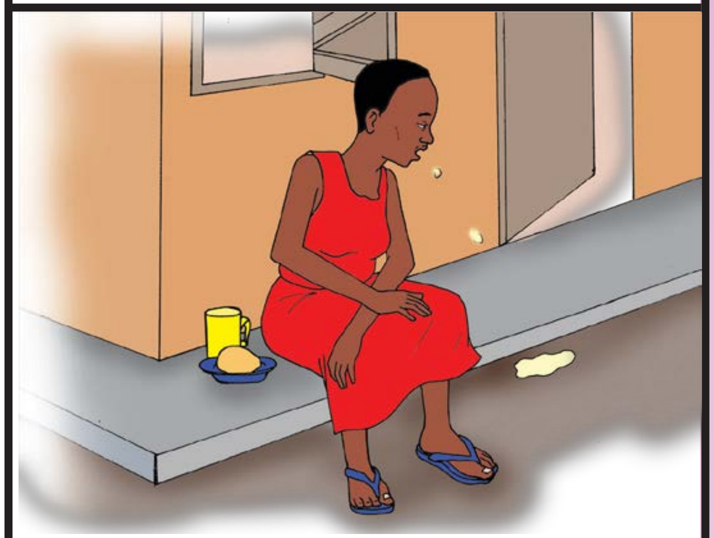

Spitting

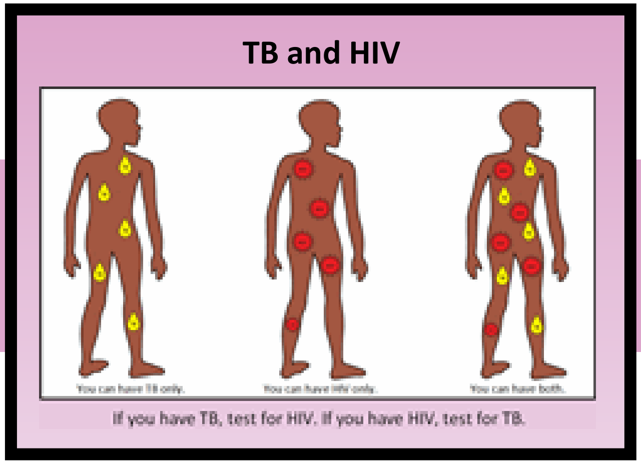

Notes to the health worker

If you know that your patient has HIV, explain the following:

- *TB speeds up the progression of HIV.*
- *HIV can make "silent" TB infections become active.*

Encourage your patients with HIV to schedule a TB test if they have not done so.

TB and HIV

OBJECTIVE:

To discuss the relationship between TB and HIV infection

TB and HIV are not the same, but it is common for people who have one to also have the other.

- You can have TB only.
- You can have HIV only.
- You can have both TB and HIV.

Half of the TB patients in Uganda also have HIV. This is because TB can easily attack people whose bodies cannot fight disease. HIV weakens the body's ability to fight disease. People with TB should test for HIV so they can get treatment early.

It's also important that people with HIV test for TB. If they have TB, they should take treatment to cure it.

TB is the most common cause of death for people with HIV.

Review Question:

Explain why someone with TB should test for HIV.

Explain why someone with HIV should test for TB.

TB and HIV

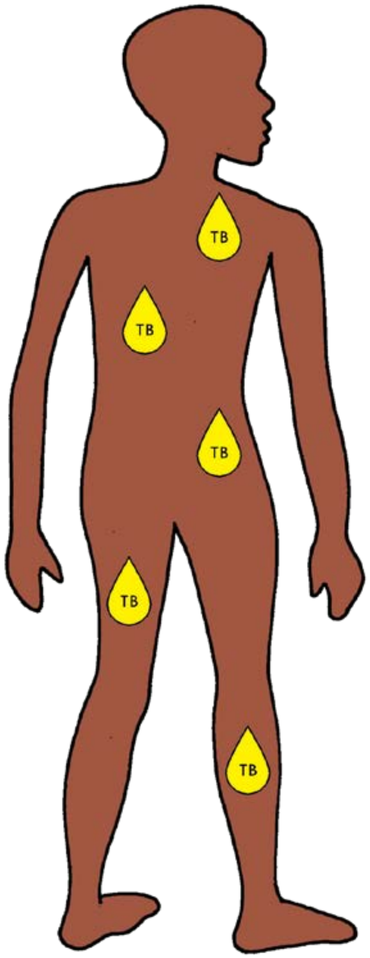

You can have TB only.

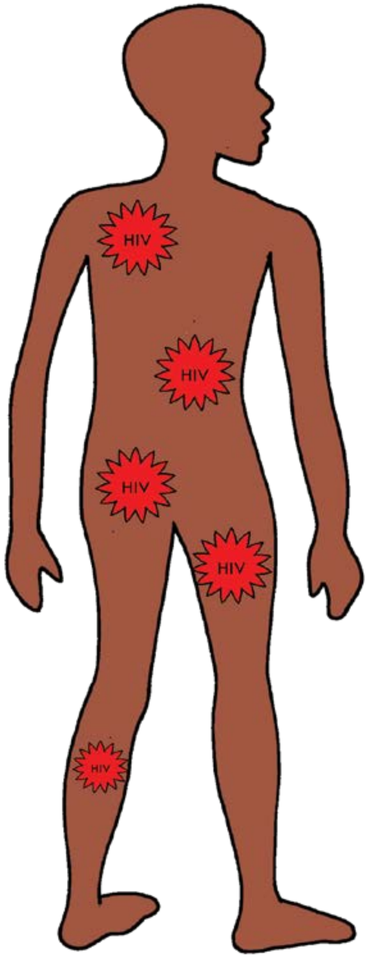

You can have HIV only.

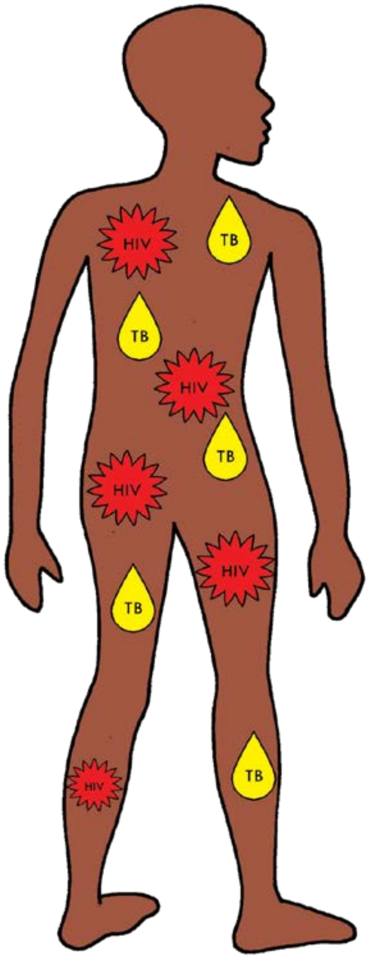

You can have both.

If you have TB, test for HIV. If you have HIV, test for TB.

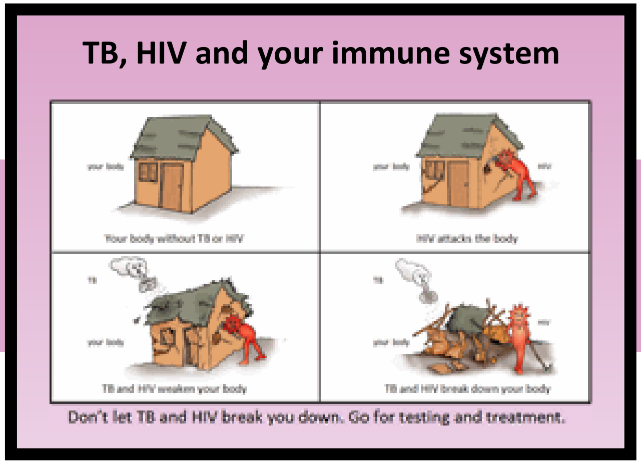

Notes to the health worker

- *Ask your patients if they understand the term "immune system." Help them understand that the immune system is a part of the body that protects them from getting sick.*
- *Make sure that your patients understand that the house in the picture represents the human body and that the walls of the house represent the body's immune system.*

TB, HIV and your immune system

OBJECTIVE:

To help TB and HIV patients understand what is happening in their bodies and why it is important to schedule tests for HIV and TB

Picture 1:

Imagine that your body is a house. The immune system in your body is like the walls that support your house and keep it standing strong.

Picture 2:

When the HIV virus invades your body, it slowly attacks your body's immune system. Imagine someone breaking off pieces of your house. Over time your house will become weak. That is what happens when HIV is inside your body breaking down your immune system. Your body becomes weak.

Picture 3:

What happens if your house is already damaged and a strong rainstorm comes? When TB attacks a body that is already weakened by HIV, it is like wind and rain pounding a damaged house.

Picture 4:

If you don't repair a damaged house, what happens? It collapses. TB and HIV together are a much greater threat to your health than they are alone. That is why it is very important for you to test for both as soon as possible. If you know that you have TB or HIV, you can repair your body before it is too late.

Review Question:

What can you do to protect your body so that it stays strong and does not break down?

TB, HIV and your immune system

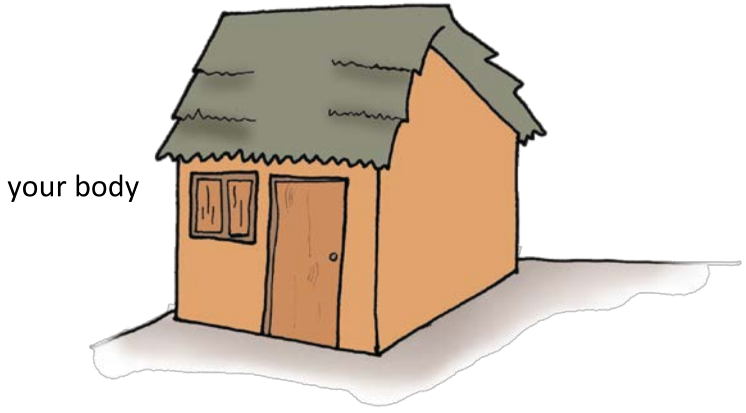

Your body without TB or HIV

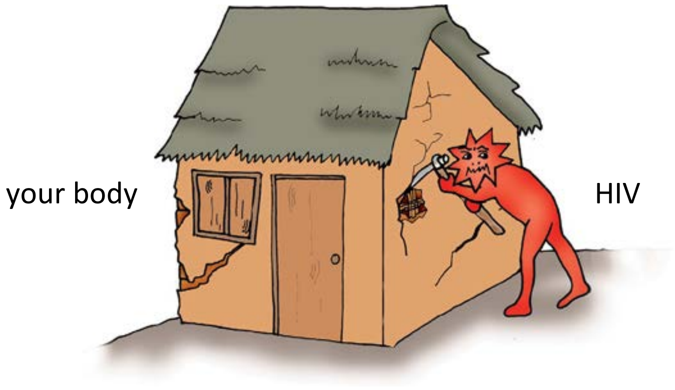

HIV attacks the body

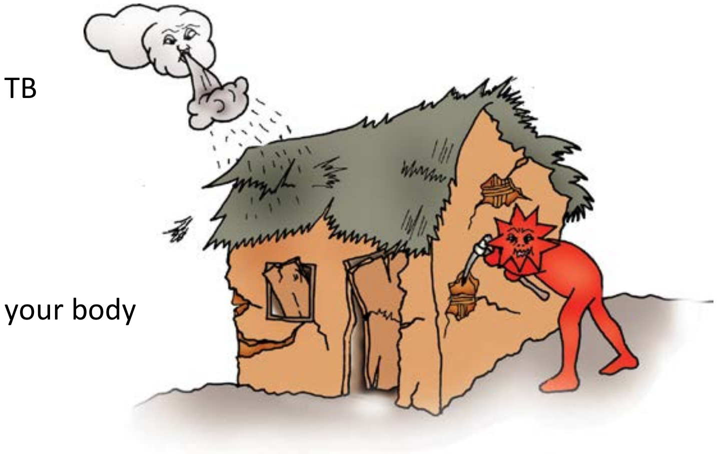

TB and HIV weaken your body

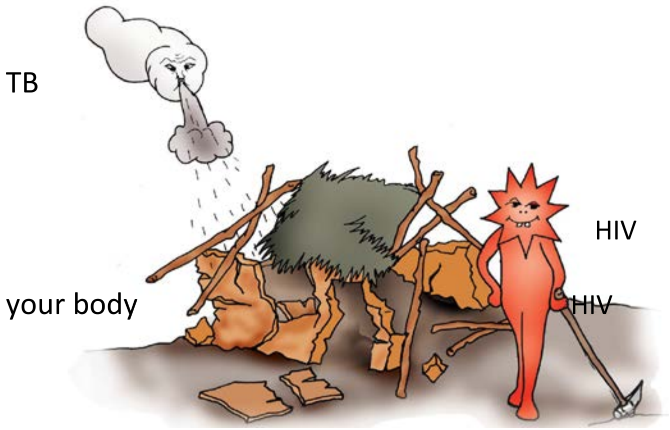

TB and HIV break down your body

Don’t let TB and HIV break you down. Go for testing and treatment.

Where do you go for TB and HIV testing?

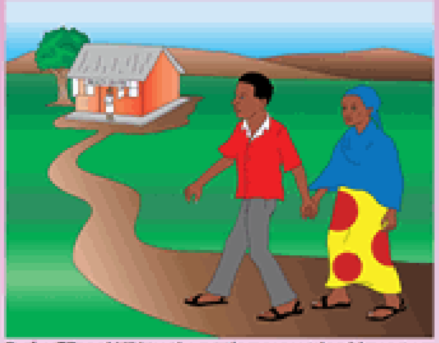

Go for TB and HIV testing at the nearest health centre.

Where do you go for TB and HIV testing?

OBJECTIVE:

To help patients understand where they can test for TB and HIV and why it’s important

Go to your nearest health centre to test for TB or HIV.

TB and HIV testing are free at government health centres.

Knowing if you have TB or HIV will help you to get treatment. It will also help prevent spreading TB or HIV to others.

Review Question:

Where can you go for free TB and HIV tests?

Notes to the health worker

Be sure that you know where to refer your patients for TB and HIV testing.

You should tell them the following:

- *The name of the testing facility*
- *The days and hours that it is open for testing*
- *Directions to the testing facility*
- *Any other relevant information that will help your patient get tested*

Where do you go for TB and HIV testing?

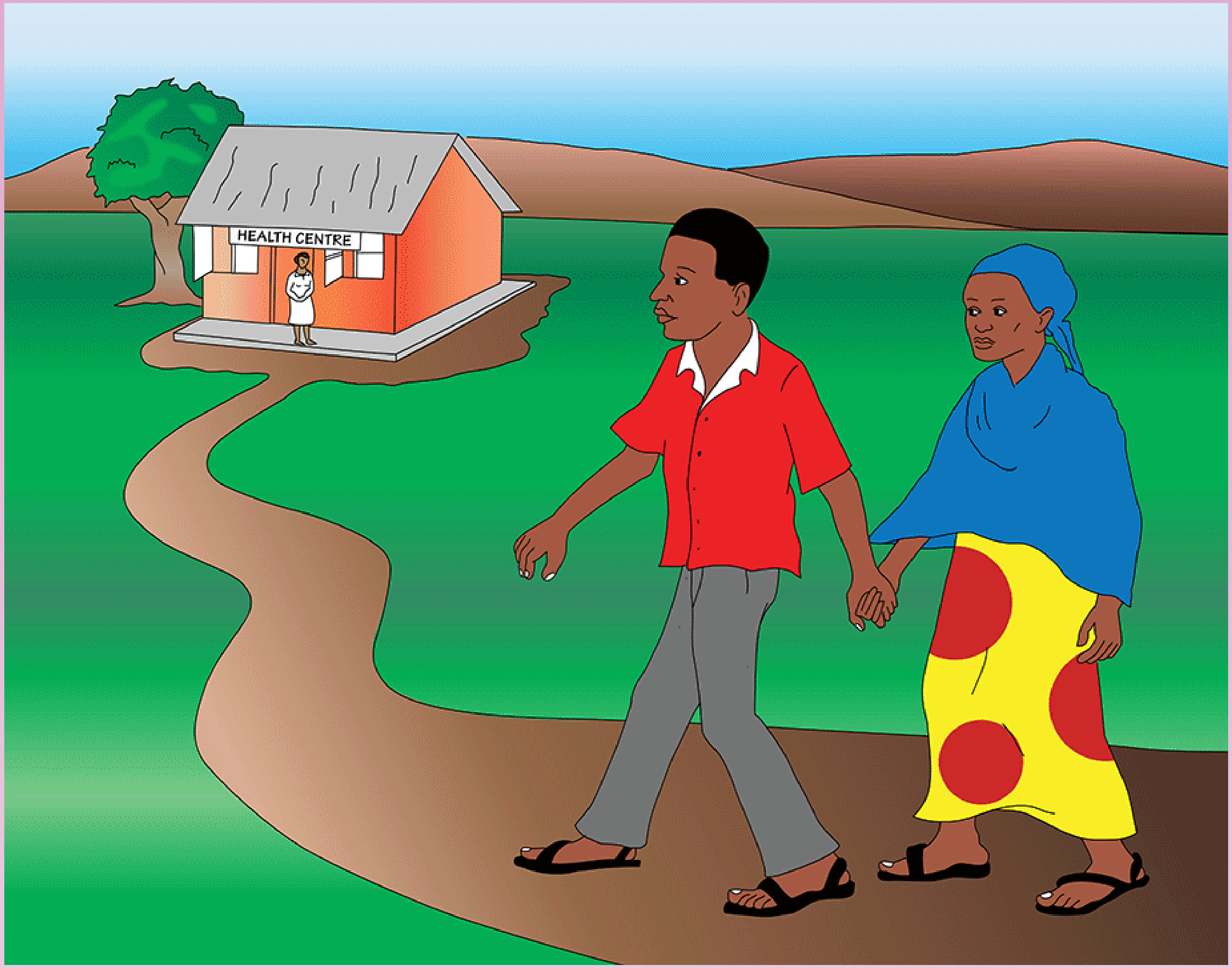

Go for TB and HIV testing at the nearest health centre.

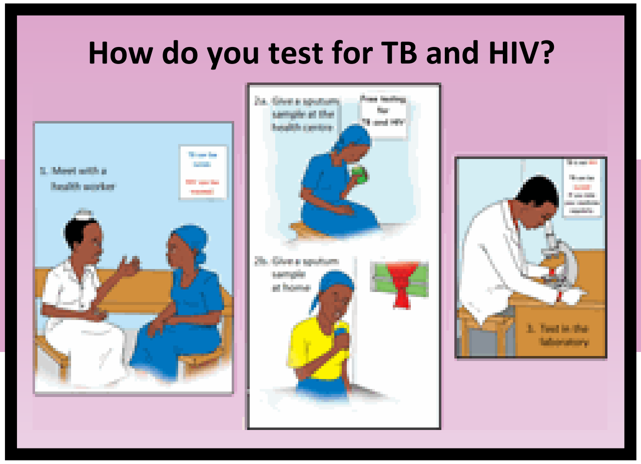

How do you test for TB and HIV?

OBJECTIVE:

To explain the process of testing for TB

Notes to the health worker

Reassure your patient that taking a test for TB or HIV is nothing to fear. Ask if they have any questions about the tests.

Picture 1:

When you go for testing you will meet with a health worker who can give you more information about TB and HIV.

Picture 2:

If you are testing for TB, you will be asked to give 2 sputum samples. Sputum is the mucus that you spit out when you have a cough. You will collect these samples in a cup provided by a health worker. You should collect the samples within 24 hours using the “spot-morning” method, which is:

- **Sample a:** At the health centre
- **Sample b:** At your home the next morning after waking up and before eating or drinking anything

Picture 3:

After giving your sputum samples, trained laboratory personnel will look at them through a microscope. If you have TB, they will be able to see the TB germs through the microscope. Laboratory personnel can also find TB in sputum by using a GeneXpert machine.

If you are testing for HIV, the lab will take a small sample of your blood. The HIV test takes only a few minutes and you will get your results the same day.

Review Question:

Describe the steps you must take to test for TB.

How do you test for TB and HIV?

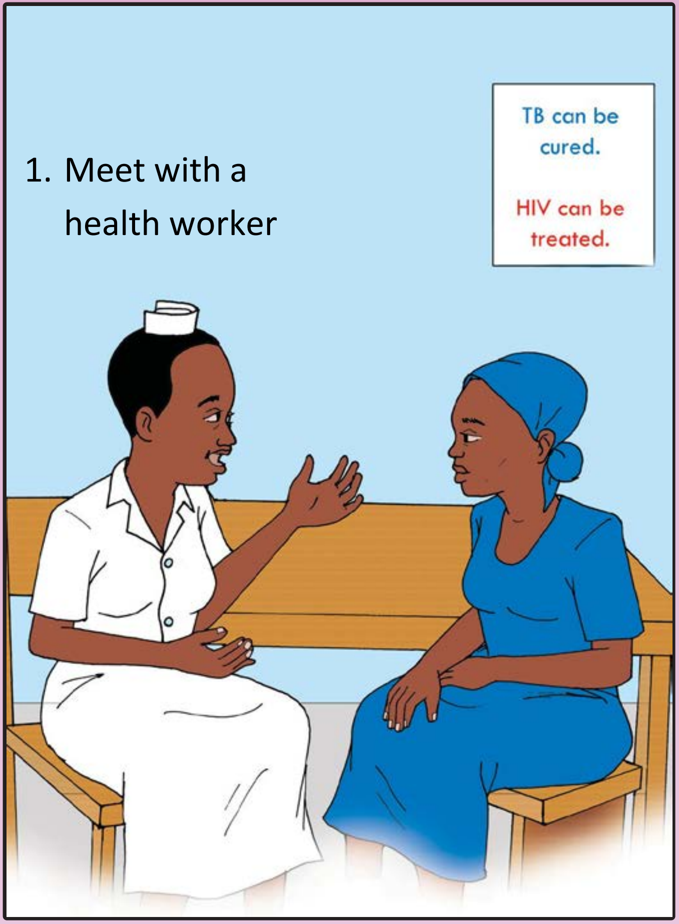

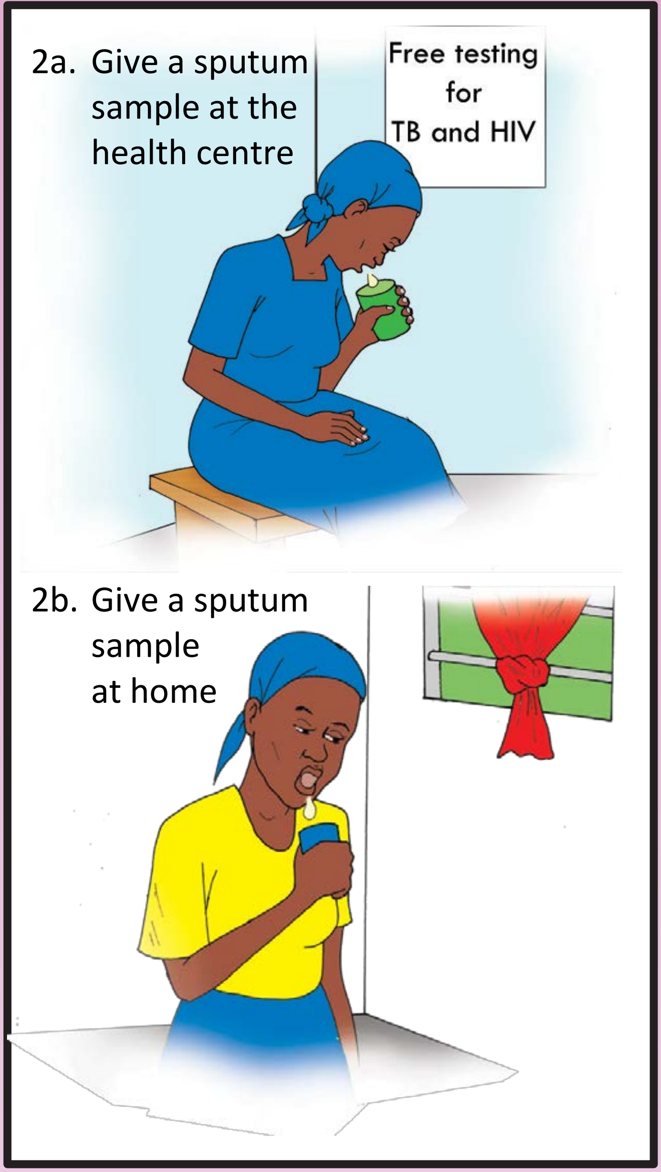

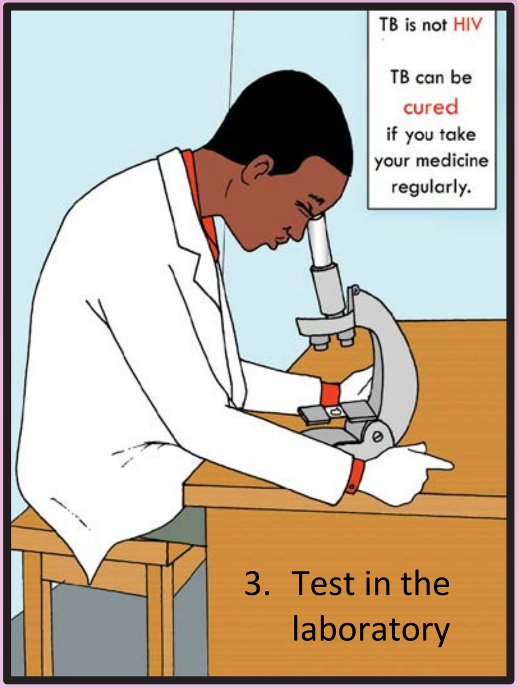

Complete your TB treatment.

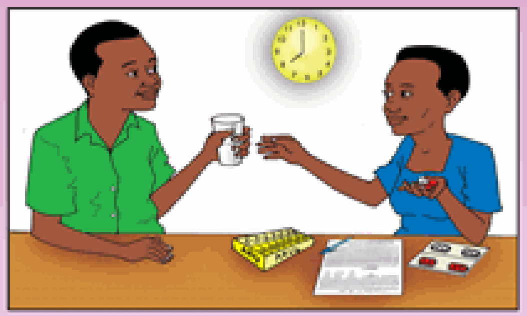

Take all your TB and HIV drugs on time each day.

Complete your TB treatment.

OBJECTIVE:

To explain why it is important for patients to adhere to their medication

TB can be cured if treated properly.

You will have to take TB treatment every day for 6 to 8 months.

Even if you feel better after a few days of TB treatment, you must take the drugs for the full time period if you want the disease to be cured.

If you stop TB treatment before 6 to 8 months, the following can happen:

- The cough that had stopped, can start again.
- Your TB becomes harder to cure.
- You spread TB to others.
- You can die early.

You should start HIV treatment in the first 8 weeks of starting treatment for TB.

HIV is not curable, but proper treatment will slow down the speed of HIV reproduction and keep your body strong.

If you have TB, HIV, or both, it is very important to eat a balanced diet.

Review Question:

Why is it important to take your TB or HIV medicine properly?

Notes to the health worker

- *The Ministry of Health adopted the **direct observed therapy, short course** (DOTS) as the most effective way of controlling and curing TB.*
- *Under DOTS, a **treatment supporter**, usually a family member, administers TB medicine to the TB patient and directly observes the patient taking that medicine each day. In the picture, the man in the green shirt is the woman's treatment supporter.*
- *The health worker helps organize a treatment supporter for each TB patient. The treatment supporter must be convenient and acceptable to the patient.*
- *The treatment supporter watches the patient swallow the TB medicine each day for the full period of treatment to ensure adherence.*

Complete your TB treatment.

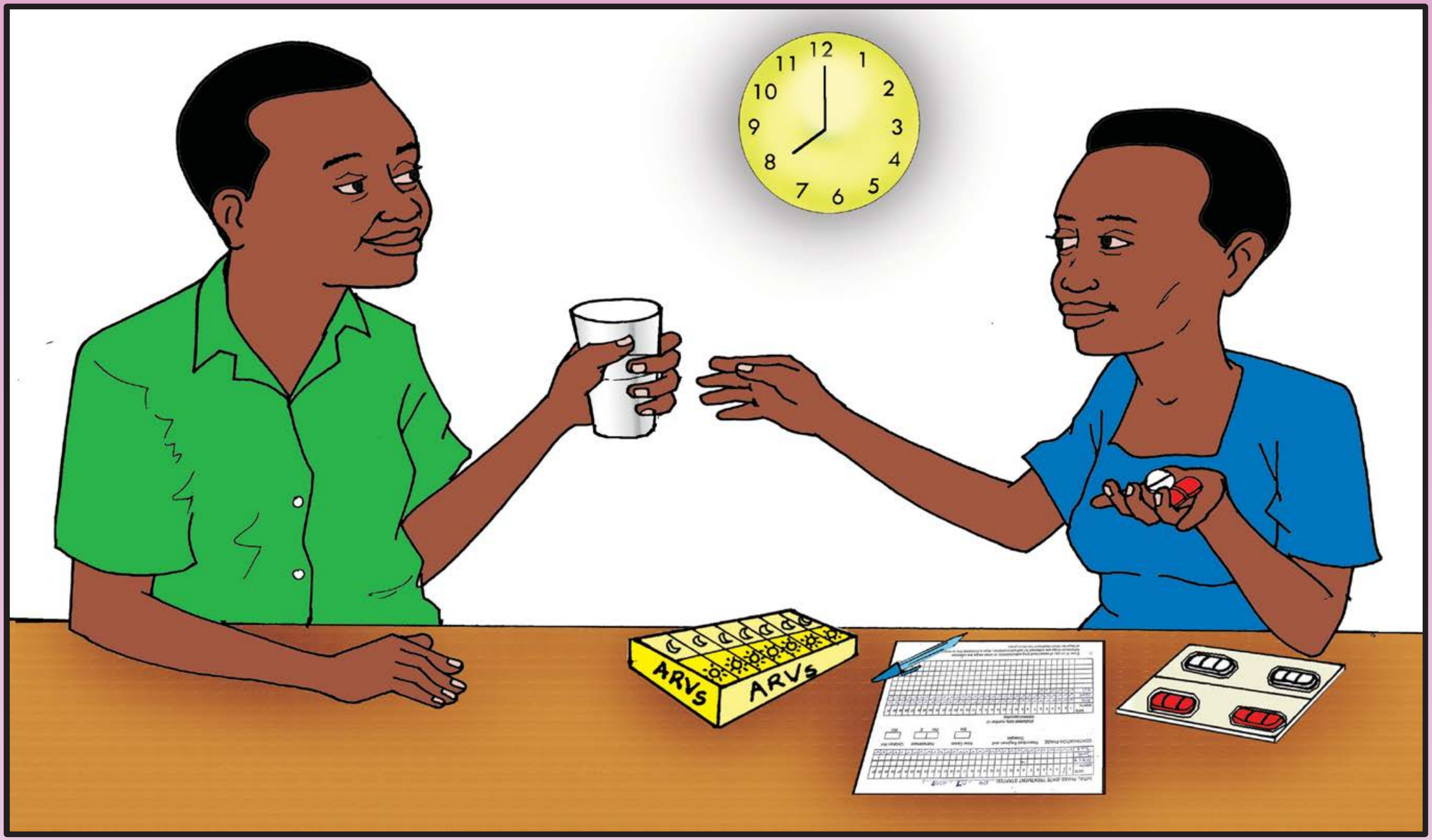

Take all your TB and HIV drugs on time each day.

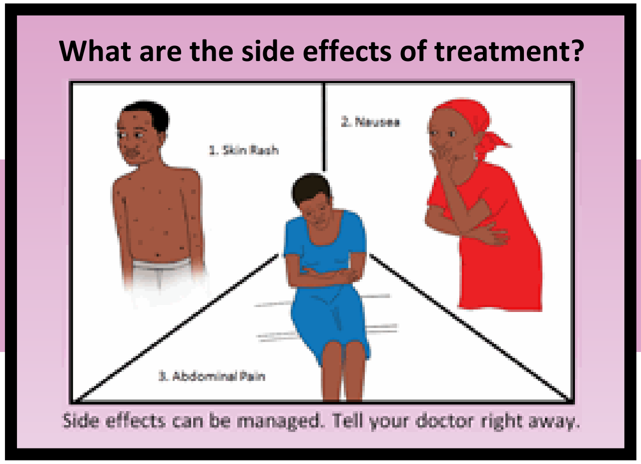

Notes to the health worker

Ask your patients if they have any questions or concerns about TB or HIV treatment.

Reassure them that their doctor or health worker can help them if they are having side effects.

What are the side effects of treatment?

OBJECTIVE:

To discuss the side effects of TB and HIV treatment

Some people have side effects from taking TB and HIV drugs.

Three examples are shown on the chart.

1. Skin rash

2. Nausea

3. Abdominal pain

Other side effects include:

- Yellowish skin or eyes
- Fever for 3 or more days
- Dry mouth
- Headache

You should not stop treatment if you have side effects. Talk to your doctor or health worker. They can help you if you are having side effects.

Review Question:

What are some of the side effects of TB or HIV treatment?

What are the side effects of treatment?

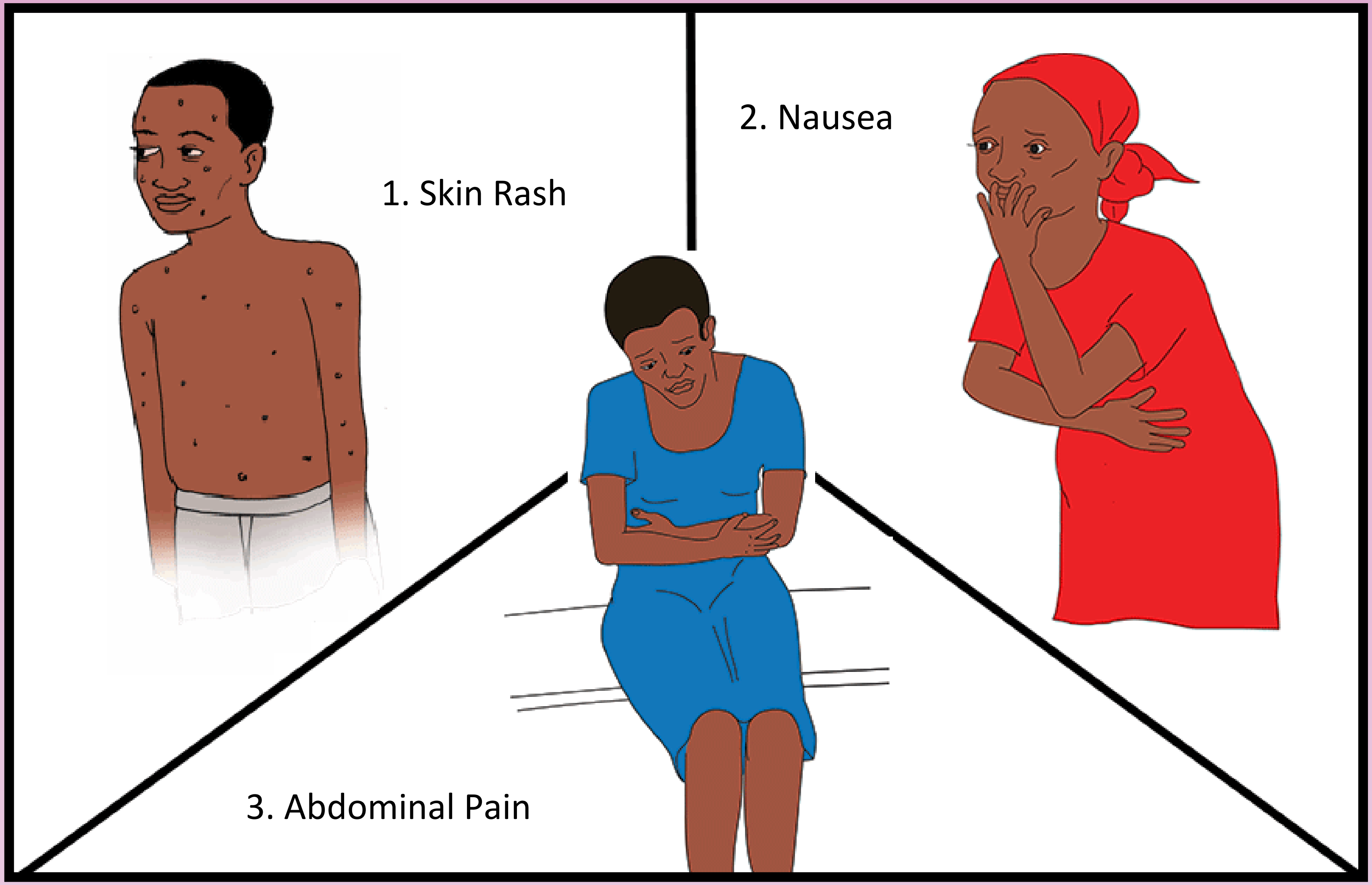

Side effects can be managed. Tell your doctor right away.

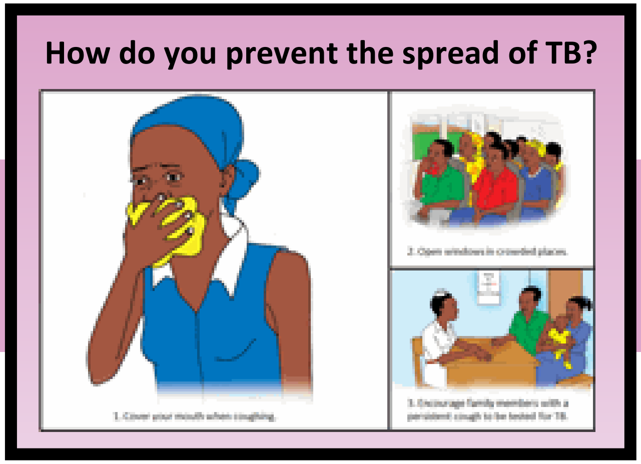

How do you prevent the spread of TB?

OBJECTIVE:

To discuss ways of preventing the spread of TB

TB patients can help stop the spread of TB.

TB patients should:

1. Cover their mouth with a handkerchief when coughing
2. Open windows in the home and in crowded places
3. Encourage family members to test for TB if they have a cough lasting 2 or more weeks

The best way to prevent the spread of TB is to treat and cure it, so take your TB treatment as prescribed and complete your TB treatment.

Encourage friends and family members to test if:

- They have been coughing for 2 or more weeks
- You suspect they may have TB
- They have HIV with TB symptoms
- They stay with a TB patient

Review Question:

What can a TB patient do to prevent spreading the disease?

How do you prevent the spread of TB?

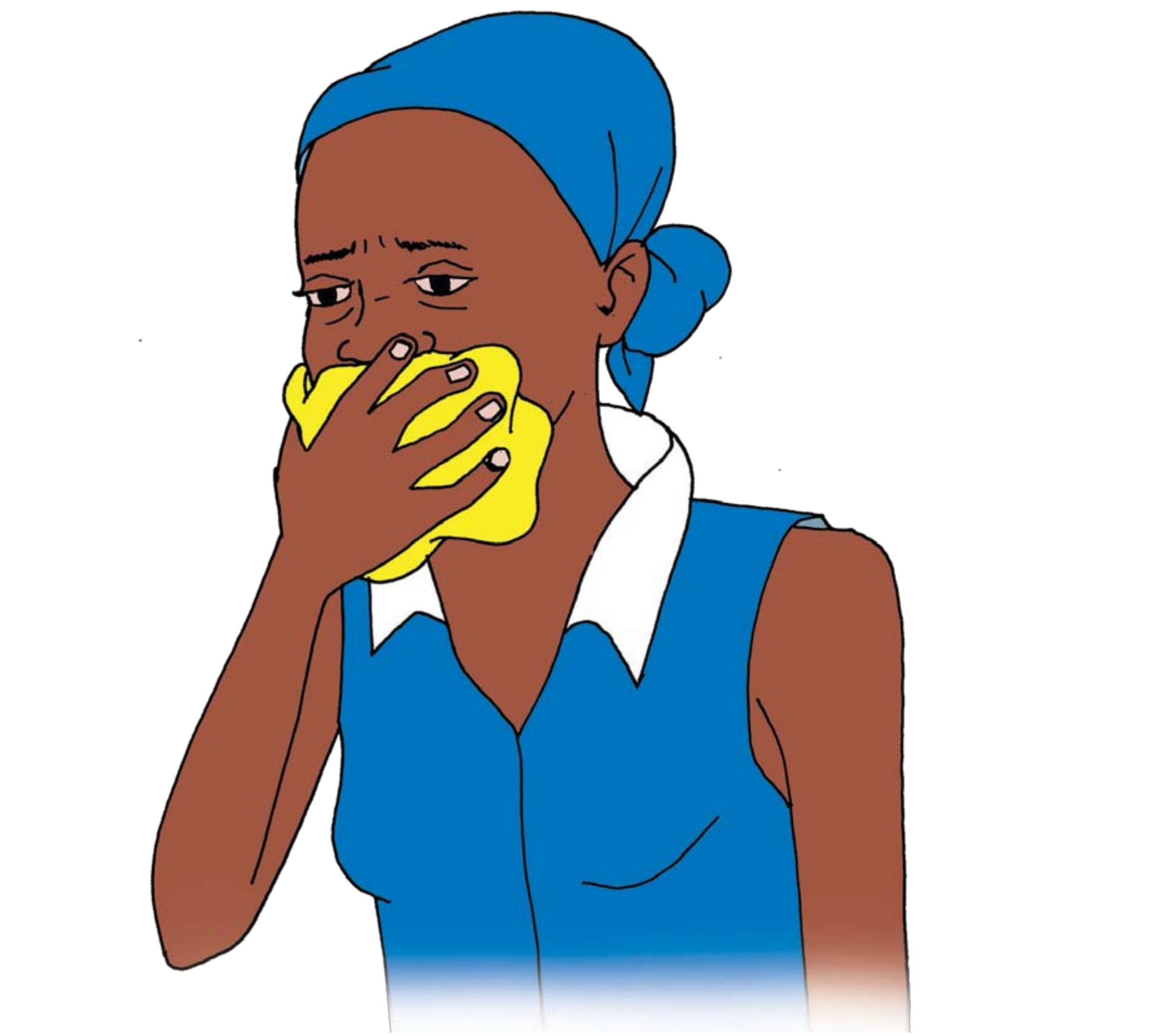

1. Cover your mouth when coughing.

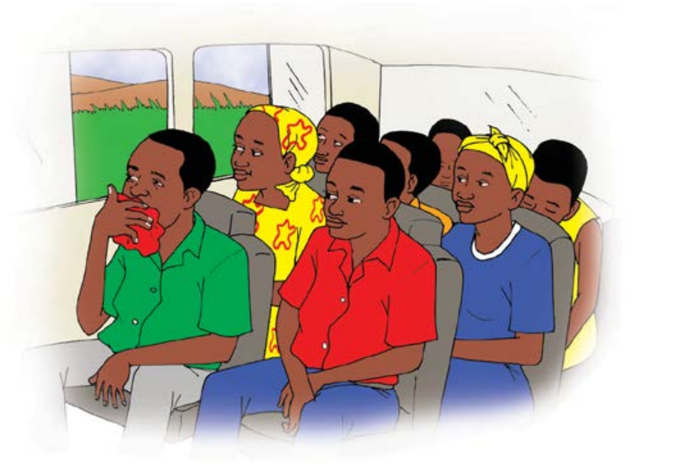

2. Open windows in crowded places.

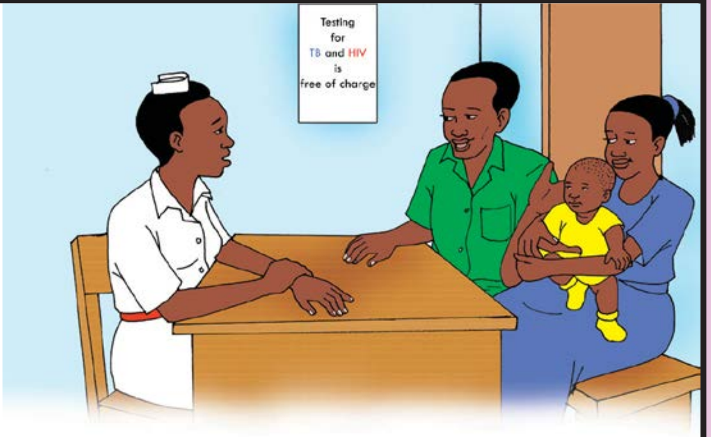

3. Encourage family members with a persistent cough to be tested for TB.

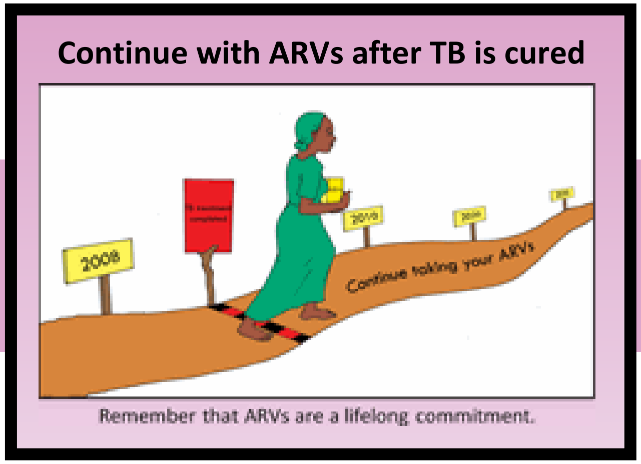

Continue with ARVs after TB is cured

OBJECTIVE:

To emphasize the importance of continuing with ARVs after TB treatment

Patients who have both TB and HIV can become healthy again. With proper treatment, TB can be cured after 6 to 8 months.

Even though HIV cannot be cured, you can become healthy after recovering from TB if you take your ARVs on time, every day for the rest of your life.

Review Questions:

How long is TB treatment?

How long do you have to take your ARVs?

Notes to the health worker

- *Help your patients understand that the road in the picture represents their life. Point out the signs showing the years.*
- *Explain that the woman in the picture has just completed her TB treatment, but she will continue to take ARVs for the rest of her life.*
- *This is the last chart. Ask your patients if they have any more questions or concerns.*
- *Remind patients where they can go for both TB and HIV testing and treatment.*

Continue with ARVs after TB is cured

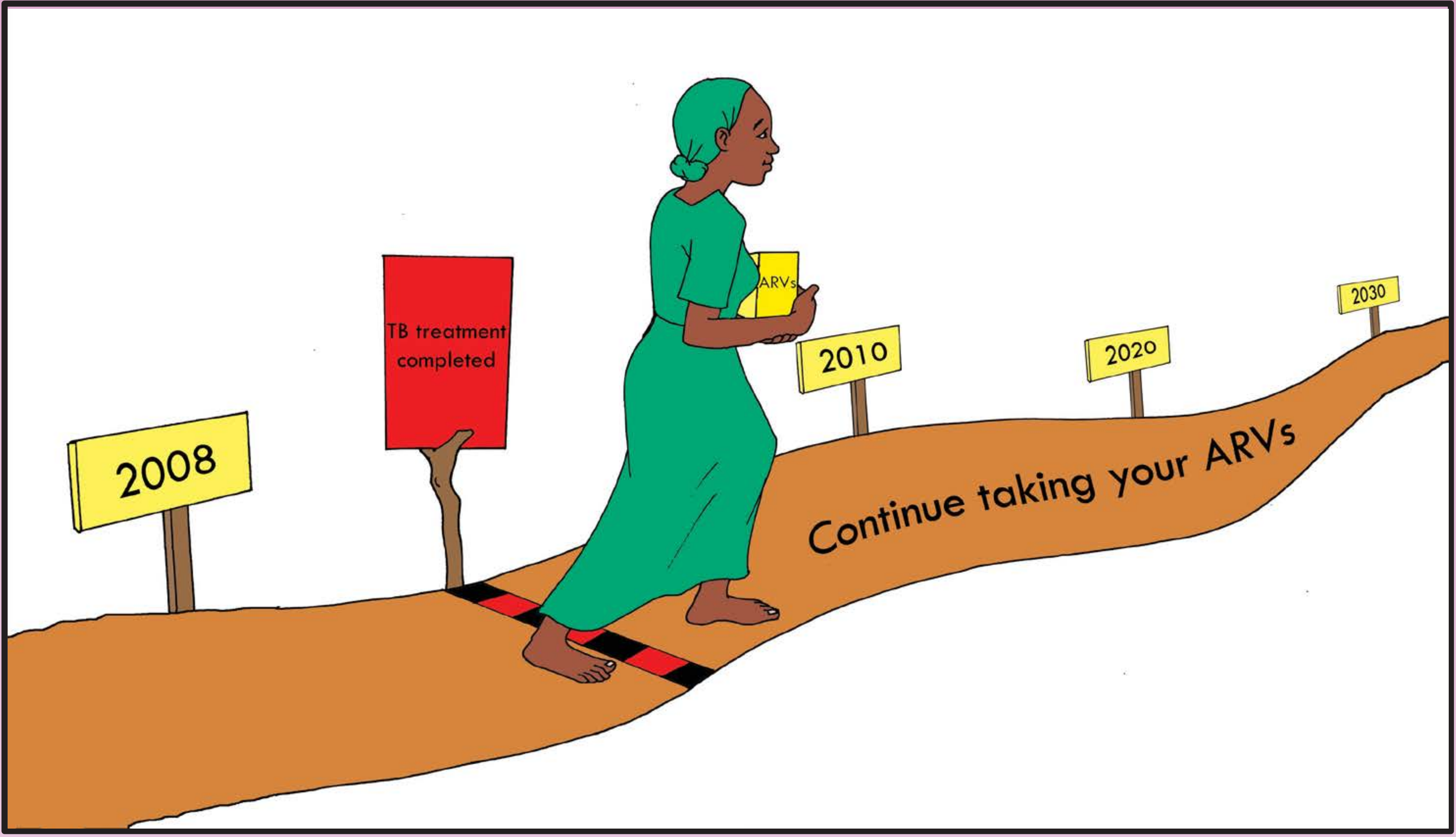

Remember that ARVs are a lifelong commitment.

TB and HIV Counseling Flipchart

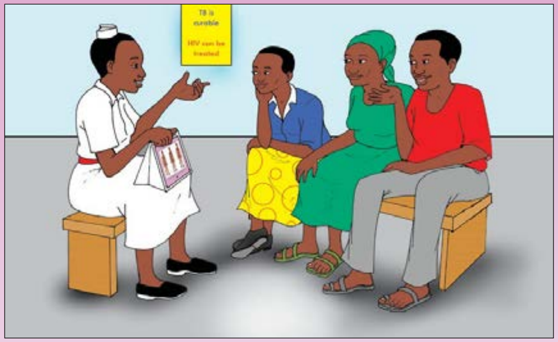

The design and printing of this flipchart was made possible by the generous support of the American people through the President's Emergency Plan for AIDS Relief (PEPFAR) and the United States Agency for International Development (USAID). The contents are the responsibility of TRACK TB, Uganda's Ministry of Health, and FHI 360, and do not necessarily reflect the views of PEPFAR, USAID, or the United States Government.

Designed for Uganda's Ministry of Health with technical assistance from Health Communication Partnership (HCP) and funding from the United States Agency for International Development (USAID).
