# Supplementary material for: Effectiveness of routine tuberculosis education in a high-burden setting: A prospective observational cohort study
Source: PLoS One. 2026 Mar 18;21(3):e0344250. doi: 10.1371/journal.pone.0344250 (PMC12998860; doi:10.1371/journal.pone.0344250)
Supplement: S2 Appendix — (PDF) [file pone.0344250.s003.pdf]

S2 Appendix. Individual Question Scores

Question	Pre-Test	Post-Test	Two-Week	Two-Month	Five-Month
TB attacks the lungs	85%	96%	99%	100%	98%
TB can attack other parts of the body outside of the lungs	32%	67%	61%	42%	62%
Everyone who is exposed to TB germs becomes ill	31%	31%	25%	8%	18%
Warning sign of TB: A cough that does not go away for two weeks	87%	96%	99%	97%	100%
Warning sign of TB: Loss of weight	90%	90%	97%	94%	98%
Warning sign of TB: A cough that goes away after a few days	81%	92%	96%	83%	84%
Warning sign of TB: General weakness	55%	64%	74%	58%	75%
Warning sign of TB: Vomiting	44%	56%	47%	42%	46%
TB spread: Through drinking water	31%	41%	28%	15%	15%
TB spread: Through the air	97%	99%	97%	97%	98%
TB spread: Through food	39%	47%	42%	17%	38%
Unprotected sex can spread TB	51%	56%	60%	55%	71%
If you stop treatment before the full course of therapy, your TB becomes harder to cure	96%	96%	100%	100%	100%
How often can TB be cured if treatment is started in time?	91%	100%	100%	100%	100%
How are TB germs released?	58%	86%	79%	67%	71%
If you breathe in TB germs, where do they settle and grow?	45%	71%	65%	55%	71%
Can you have HIV only (without TB)?	92%	99%	99%	99%	98%
Can you have TB only (without HIV)?	90%	99%	100%	99%	100%
How can you stop the spread of TB?	47%	82%	79%	77%	80%
If you have TB, how long do you take the medication?	53%	91%	93%	97%	100%
When can you stop taking the TB medication?	50%	89%	93%	97%	96%
If you stop taking the TB medication before the treatment period is finished, what might happen?	94%	96%	99%	100%	100%
Name two potential side effects of TB treatment	6%	67%	61%	47%	55%
What should you do if your TB medication gives you nausea?	23%	42%	53%	35%	46%
What should you do if your TB medication gives you joint pain?	30%	56%	60%	35%	44%
What should you do if your TB medication gives you yellow or red eyes, too much	55%	89%	89%	94%	80%

vomiting, intense body rash, or issues with sight?					
When should you come to the clinic for your next appointment?	10%	95%	93%	96%	98%
What do your TB medications look like?	26%	96%	99%	100%	98%
When do you take your TB medications?	27%	99%	97%	99%	100%
After starting the medication, how long does it usually take to start feeling better?	31%	55%	65%	46%	36%
