# Supplementary material for: Effectiveness of routine tuberculosis education in a high-burden setting: A prospective observational cohort study
Source: PLoS One. 2026 Mar 18;21(3):e0344250. doi: 10.1371/journal.pone.0344250 (PMC12998860; doi:10.1371/journal.pone.0344250)
Supplement: Attachment — Submitted filename: Reviewer Recommendation and Comments.docx [file pone.0344250.s004.docx]

**Reviewer Recommendation and Comments for Manuscript Number PONE-D-25-37051**

**Comments on the Abstract**

**Comment 1**

In the Abstract the authors have an inconsistence between the title of the manuscript as “efficacy” while the information in the background refers to effectiveness (line 4). I recommend the authors stick with effectiveness that best suits the methodology of this study.

**Comment 2**

The abstract title is about education, but the authors have included counselling in line which a different concept. I recommend the authors stick to the education aspect in order not to confuse the readers.

Introduction

**Comment 3**

The authors explain the gaps in the current approach and propose to explore studying the specific TB knowledge aspects influenced by education in the routine setting. The authors however again refer to “efficacy” of intervention in the routine setting in line 46. In routine setting the authors could not control study events, the appropriate term should be “effectiveness”

Materials and methods

**Comment 4**

The authors revise appropriately to replace the wording of efficacy with effectiveness in line 53, 60

**Comment 5**

Primary Objective 1 focuses on TB knowledge. The authors have defined TB knowledge to encompass TB disease and treatment constructs. According to line 38 it suggests that TB knowledge is a combination of TB disease and TB treatment. The authors further define TB specific knowledge constructs. This creates confusion since the conclusions focus on the TB specific knowledge constructs. I recommend that the authors clearly differentiate between these two concepts and report on results aligned with the TB knowledge as stated in the objectives .

*Development of an instrument for assessing TB knowledge*

**Comment 6**

The authors has explained how they developed the tool and provided the reference materials. I however that note that the authors considered adding counselling to the tool whereas the objectives are focused on TB education. The authors should clarify to what extend counselling was conducted and how that influenced the TB education for the clients

**Comment 7**

I have reviewed the S1 appendix and found that it contains basic questions understandable to the clients. The authors, however, are using complex terms such pathogenesis, treatment mechanisms etc which terms are not a representation of the S1 Appendix nor the description or expectation of the client to be given to the clients. I recommend authors use terms consistent with the questions asked such as for TB disease (TB disease, TB symptoms, TB transmission, TB prevention, TB/HIV relationship) and for TB treatment (TB medicines, how to take TB medicines, Response to TB treatment, TB treatment follow up visits, adherence to treatment).

Procedures

**Comment 8**

The authors explain how the education was conducted. The method of delivery was not the same and the materials used to deliver were not the same for all the participants. For clarify to the readers, the authors should explain the factors that determined the different delivery methods and discuss their impact on the results of this study.

Sample size estimates

**Comment 9**

It is not clear why the authors did not provide or make a statement of why the objective of education to increase TB knowledge was not used to compute sample size. It is important that the readers are sure about the power to make the conclusions. The authors indicate TB knowledge is a combination of TB disease and TB treatment knowledge. The authors should demonstrate and justify that the sample size used was the highest and therefore provides power for all objectives. I have reviewed the references for sample size assumptions and find no clear linkage. Since sample size is critical for validity of conclusions, I recommend that the authors revisit the sample size computation approach and assure the readers about the validity of the conclusions.

Statistical analysis

**Comment 10**

The authors should provide the scientific merit of weighting responses to questions. I think a client would have knowledge or not to a particular question. For the interest of the readers, the authors should provide the scores for the questions in S1 Appendix and provide how the percentage score for TB knowledge was determined for each client. It is not clear why the statistical analysis was not focused on the TB knowledge increase as the outcome but rather the nonadherence. This approach confuses the readers and makes it difficult to relate the study objectives with the results and conclusions. The authors should provide more clarity how the scores were generated and how there related to the percentages.

Results

**Comment 11**

Line 166-195; This section on results is unclear. the authors should display the results for easy understanding and following of the readers. I recommend the results are presented in accordance with the objectives presented. The disease specific aspects are secondary results and should be provided as such. The clients reaching adequate knowledge are not shown in the table and yet this is central to the study. I recommend these results are clearly shown to the readers.

**Comment 12**

Line 211-225; The authors intend to report on TB knowledge and nonadherence in this section. There is no result on TB knowledge and nonadherence instead of the authors report on the domains. This is inconsistent with the set-out objectives and consequently lack of power to make any valid conclusions. I recommend the authors first display results aligned with objectives and can then display other sub analysis results.

**Comment 13**

Line 235- 242; the authors intend to report on the association between TB knowledge and treatment outcomes. The authors, however, selectively report of a few domains. The overall TB knowledge should be the predictor variable rather than some domains. Since the study was not powered to conclude on association between type of education delivery method and outcome, it is for the authors to acknowledge lack of power to make the conclusion.

**Comments 14**

Line 304-323; the authors have stated the limitation to this study. However uncertainty about the power calculation based on nonadherence while the primary study was TB knowledge increase should be made clear so the readers put the results of this work in proper context.

**Comment 15**

Line 326; the conclusion statement implies that routine education sustains knowledge. This is not what the methodology performed. Routine education was only provided at baseline and therefore there is no evidence to suggest it sustained the knowledge. I recommend the authors revise the sentence.
